# Mitigation of lead stress in *Zea mays* L. plants: the role of *Trichoderma harzianum* Zag-1 in enhancing antioxidant defense and physiological stability

**DOI:** 10.1186/s12870-026-09299-8

**Published:** 2026-06-23

**Authors:** Asmaa S. Taha, Shereen A. Soliman, Rabab A. Metwally

**Affiliations:** Botany and Microbiology Department, Faculty of Science, Zagazig University, Zagazig, 44519 Egypt

**Keywords:** Heavy metals, Lead (Pb), *Trichoderma harzianum*, Maize, Relative water content, Stress marker, Proline.

## Abstract

**Background:**

Lead (Pb) is a highly toxic non-essential element, whose toxicity has been documented globally due to its disruption of critical plant functions and its effect on overall yield, ultimately entering the food chain. Beneficial microbes such as *Trichoderma* sp. play a pivotal role in promoting plant growth, especially under heavy metal stress.

**Results:**

In this study, a novel fungal bio-inoculant, *Trichoderma harzianum* isolate Zag-1, was utilized both in vitro and in a pot experiment to alleviate Pb-induced stress. According to in vitro preliminary findings, *T. harzianum* Zag-1 could tolerate Pb (II) up to 1500 ppm on potato dextrose broth (PDB) medium and had a maximum removal efficiency of 70.40% at 500 ppm. Alongside, *T. harzianum* was used to improve the growth of maize plants at two levels (low: 500 ppm and high: 1000 ppm), and the results indicate that both levels negatively affect its growth and physiological traits. Exposure to 1000 ppm Pb severely inhibited maize development, resulting in reductions in shoot height (13.04%), root length (28.17%), shoot and root fresh weights (Fwt) (34.03% and 20.74%), and dry weights (Dwt) (41.79% and 15.15%), respectively, as compared to the controls. Furthermore, Pb (II) stress at 1000 ppm significantly depleted photosynthetic pigments (chlorophyll a, Chl b, carotenoids, and total pigments reduced by 52.33%, 47.37%, 58.62%, and 52.75%, respectively) and impaired water status, causing a 27.26% reduction in relative water content (RWC) in comparison to non-stressed plants. Furthermore, in Pb-stressed plants (1000 ppm), oxidative stress markers including membrane leakage (ML), malondialdehyde (MDA), and hydrogen peroxide (H_2_O_2_) were increased significantly by 148.58%, 105.31%, and 50.03%, respectively, as compared to non-stressed control plants. However, the inoculation of plants with *T. harzianum* Zag-1 increased Fwt (17.96%) and Dwt (17.00%) of biomass, Chl a (69.77%), Chl b (52.63%), RWC (2.84%), and membrane stability index (MSI, 10.89%) in comparison to their control counterparts. *T. harzianum-*inoculated plants had 12.36%, 45.65%, 37.65%, and 4.56% higher soluble protein, proline, carbohydrates, and glycine betaine concentrations, respectively, than non-inoculated controls at 1000 ppm of Pb. However, at 1000 ppm Pb, ML, MDA, and H_2_O_2_ contents decreased by 23.59%, 40.36%, and 25.41%, respectively, compared to *T. harzianum*-free plants. Results on antioxidants showed that *T. harzianum* inoculation led to a significant rise in enzyme activities (10.36, 29.22, and 8.44%, for peroxidase [POD], polyphenol oxidase [PPO], and ascorbate peroxidase [APX], respectively) in plants treated with 1000 ppm of Pb.

**Conclusion:**

Remarkably, the locally isolated *T. harzianum* strain Zag-1 demonstrates a distinct and enhanced tolerance to Pb stress, coupled with a superior ability to modulate antioxidant defense, ionic homeostasis, and metabolic adjustments of maize plants under Pb stress, presenting a valuable tool for promoting climate-resilient farming in Pb-affected areas.

## Introduction

In response to changing environmental conditions, the issue of heavy metal (HM) in the plant–soil environment has resulted in a significant decrease of growth and development of major crops worldwide [1]. HM contamination has increased due to mining, industrial output, municipal sewage, rapid economic growth, and agricultural pollutants [2], and the agricultural output is becoming seriously threatened by this pollution [3–5] and further endangers human health. One of these HMs is Lead (Pb), one of the major worldwide issues because it has widespread toxic effects on both living things and the larger ecosystem [6, 7]. The Pb contamination is continuously increasing through a variety of anthropogenic and natural activities and due to its high industrial demand [8]. Being a non-essential element, it negatively affects plant life, which directly lowers soil fertility and persists in all environmental compartments (soil, water, and air), all of which together pose an urgent ecological threat. It is commonly reported that the Pb concentration in contaminated soils ranges from 400 to 800 ppm, but in industrial areas, this value frequently increases to 1000 ppm [9]. A detrimental ecological cascade is started when Pb is present in the soil that changes in the diversity and metabolism of microorganisms, which subsequently lowers microbial growth and enzymatic activities [10]. Furthermore, plant growth and agricultural productivity are eventually hampered by this loss [11].

Food security and sustainable crop production are under risk due to the growing problem of Pb pollution in agricultural soils [12]. Maize (*Zea mays* L.), a cereal grain in the Poaceae family, is among the most extensively grown crops in the world and occupies third in the worldwide among crops in terms of the cultivated area [13, 14]. A Staple crop grown all over the world, maize is especially vulnerable to Pb toxicity, which reduces its yields and growth [12]. Consequently, there has been a growing demand for developing methods to lessen crop accumulation, including chemical, physical, and biological technologies [15, 16]. Among these, biological approaches received considerable attention because they are based on sustainable, economically efficient methods [17, 18]. The efficacy of helpful microorganisms in reducing stress may provide the opportunity for some novel approaches to improve maize crops’ resistance against HMs stress, ensuring food security and boosting agricultural sustainability [19, 20]. Species within the genus *Trichoderma* (family Hypocreaceae) are extensively studied as viable and environmentally friendly methods for enhancing plant development and alleviating biological and environmental stresses such as salinity, drought, and HM [21–24]. *Trichoderma* species are isolated from various environments, since they are able to establish endophytic relationships with plants [25] and can be utilized to mitigate HM toxicity while stimulating plant growth [26]. *Trichoderma* sp. promotes crop growth by multiple methods, including nutrient acquisition, organic acid and siderophore production, antimicrobial metabolite generation, and stress-responsive phytohormone synthesis [27, 28]. In environments contaminated with HMs, *Trichoderma* sp. not only demonstrates high tolerance but also has the ability to accumulate significant amounts of HMs because of its capability to produce metal-chelating compounds, which play a crucial role in mitigating HM contamination, making *Trichoderma* sp. a promising eco-friendly bioremediation agent [29, 30]. *Trichoderma harzianum*, a beneficial fungus, has been extensively utilized for growth enhancement and biocontrol. Recently, it gained significant attention for the enhancement of stress tolerance in plants subjected to abiotic conditions [31].

Despite extensive evidence highlighting the role of *Trichoderma* sp. in alleviating HMs stress, current knowledge remains limited regarding strain-specific variability, particularly among locally adapted isolates and their precise mechanisms of action under Pb toxicity. Most previous studies have focused on general stress mitigation without clearly distinguishing whether tolerance is achieved through exclusion of Pb uptake or through internal detoxification mechanisms. The functional attributes of newly isolated strains, such as *T. harzianum* Zag-1, remain insufficiently explored, especially in terms of their potential for enhanced Pb tolerance and their capacity to regulate antioxidant defense systems and metabolic adjustments. Therefore, this study addresses this gap by investigating the strain-specific responses of Zag-1 and elucidating its role in conferring Pb tolerance through integrated physiological and biochemical mechanisms. Consequently, the current study aims to evaluate the protective role of a locally isolated fungal bio-inoculant, *T. harzianum* Zag-1, in supporting the growth of *Z. mays* L. under induced Pb toxicity. The primary objective is to examine morphological and physio-biochemical attributes of *Z. mays* under this stress to provide a new biological solution in Pb stress alleviation within an agricultural system.

## Materials and methods

### Fungal material

*Trichoderma harzianum* isolate Zag-1 was previously isolated from the soil of the El-Dakahlia governorate, molecularly identified based on the internal transcribed spacer (ITS) region sequencing using ITS1/ITS4 primers, and the sequence was submitted to the NCBI GenBank database with accession number [PX649202] [28]. The pure culture *T. harzianum* Zag-1 was preserved on Potato dextrose-agar (PDA) slants, stored at 4 °C, and sub-cultured successively.

### Assessment of the in vitro tolerance of *T. harzianum* Zag-1 to different Pb(II) concentrations

#### Effect on radial growth and dry weights (Dwt)

Lead nitrate (Pb (NO_3_)_2_, 99% purity (Sigma-Aldrich, USA), was used in the present work. Active culture of *T. harzianum* Zag-1 mycelial discs (10 mm) were cultivated on sterilized PDA media supplemented with Pb concentrations of 500, 1000, 1500, 2000, 2500, 3000, and 3500 ppm and then incubated at 28 ± 2 °C. PDA media inoculated with *T. harzianum* mycelia disc without Pb (II) act as a control. The linear growth was measured 6 days after inoculation. The percent of inhibition was estimated using the following formula [32].

$$\:Percent\:of\:Inhibition\:\left(\%\right)=C-T/C\:\times\:100$$

Where *C is the radial growth of the control and T is the radial growth of the treatment.

Regarding to Dwt determination, the potato dextrose broth (PDB) media were inoculated with *T. harzianum* mycelial discs (10 mm) in 250 mL Erlenmeyer flasks containing 100 mL of PDB with varied Pb (II) concentrations (500, 1000, 1500, and 2000 ppm). Flasks containing PDB medium devoid of Pb (II) act as controls. All treatments were incubated at 28 ± 2 °C under shaking conditions (120 rpm, Model SK- 757, Lafayette, CA 94549, USA). Fungal biomass was harvested after 8 days of incubation, and their Dwts were determined.

#### In vitro Pb (II) removal and bioaccumulation assay

The culture broths that had been filtered were collected, and the residual Pb concentration was estimated *via* atomic absorption spectrophotometer (AAS, Unicam 969) at the Faculty of Veterinary Medicine, Zagazig University.

The efficacy of *T. harzianum* in removing Pb (II) was determined by the following equation [33]:

$$\:R\:\left(\%\right)=\left(\:\frac{{P}_{0}-{P}_{e}}{{P}_{0}}\:\right)\times\:\:100$$

*R is the percentage of metal removal by fungal biomass, P_0_ is the initial concentration of Pb (ppm), and P_e_ is the final concentration of Pb (ppm) in the media.

Also, the metal uptake capacity (Q), or bioaccumulation, by fungal biomass was determined utilizing the subsequent mathematical expression according to Mohsenzadeh and Shahrokhi [33].

$$\:\mathrm{Q}=\frac{\left({\:C}_{i}-{C}_{e\:}\right)\:\times\:V}{M}$$

*Q is the metal uptake (mg/g), C_i_ and C_e_ are the initial and final concentrations of Pb (ppm), V is the volume of the solution (L), and M is fungal dry mass (g).

### In vivo effect of *T. harzianum* Zag-1 on maize growth under Pb stress

#### *T. harzianum* Zag-1 inoculum preparation

To prepare spore suspensions for seed treatment, Erlenmeyer flasks containing 100 mL PDB were cultivated with the fungal strain and maintained at 28 ± 2 °C for 10 days on a rotary shaker (100 rpm). The conidial concentration was adjusted using a hemocytometer to 2 × 10^7^ CFU mL^− 1^.

### Plant growth condition and Pb stress application

The experiment was carried out at the Botany and Microbiology Department greenhouse, Faculty of Science, Zagazig University, Egypt. The maize (*Zea mays* L.) (Trihybrid 321) seeds were obtained from the Department of Plant Breeding and Genetics at the Agricultural Research Center (Giza, Egypt). The seeds were surface sterilized by immersion in 70% ethanol for 1 min, afterward 2% sodium hypochlorite for 5 min, and washed with sterilized distilled water. Sterilized seeds were soaked in *T. harzianum* suspension (10^7^ conidia mL^− 1^) for 4 h before sowing, while the non-inoculated seeds were dipped in sterilized water. Clay soil was autoclaved (121 °C; 101 kPa; 1 h for three successive days; Model MSW-101YDX, India) prior to use to eliminate native microbial communities and ensure a controlled experimental system for evaluating the specific effects of *T. harzianum* Zag-1. Although autoclaving may influence the availability of certain nutrients, all treatments were subjected to the same conditions to maintain comparability. The inoculated and non-inoculated seeds were sown in plastic pots (25 cm diam., 10 seeds/pot) containing 2.5 kg of soil (Pb content: 15 ppm). Pots were arranged in a greenhouse under natural photoperiod (25–30 °C, relative humidity 60–70%) in a randomized complete block design (RCBD) with five replicates per treatment. Also, 100 mL of fungal inoculum was added to soil during sowing in *T. harzianum*-inoculated seeds. After 10 days of sowing, this process was repeated by adding 20 mL of a *T. harzianum* suspension (10^7^ CFU mL^− 1^) around the root zones to achieve maximum inoculation efficiency. Plants treated with sterilized distilled water served as control (non-inoculated). Lead nitrate [(Pb (NO_3_)_2_, 99% purity (Sigma-Aldrich, USA)], was used as the Pb source. To ensure accurate concentrations, the correct doses of lead nitrate were weighed for each 2.5 kg soil pot, dissolved in distilled water, and administered via a single soil drench three weeks after sowing to supply the Pb stress through the soil at two different doses of 500 and 1000 ppm. The higher Pb concentrations (500 and 1000 ppm) were intentionally applied as experimental stress levels to evaluate the mitigation potential of *T. harzianum* under severe Pb toxicity. Maize plants were harvested after 15 days of HM application for examining different growth and physio–biochemical characteristics. Six treatments are as follows:

1. Control: non-inoculated and non-stressed plants.
2. *T. harzianum*: *T. harzianum-*inoculated plants without Pb stress.
3. Pb (500 ppm): non-inoculated plants exposed to 500 ppm Pb.
4. Pb (500 ppm) + *T. harzianum*: *T. harzianum*-inoculated plants exposed to 500 ppm Pb.
5. Pb (1000 ppm): non-inoculated plants exposed to 1000 ppm Pb.
6. Pb (1000 ppm) + *T. harzianum*: *T. harzianum*-inoculated plants exposed to 1000 ppm Pb.

### Measurements

#### Characterization of phenotypic parameters

After the Pb stress period, the *Z. mays* plants from all treatment were collected and cleaned with tap water and their shoots and roots were evaluated separately in order to determine their dry (Dwt) and fresh weights (Fwt). For Dwt measurements, shoot and root samples were oven-dried at 60 °C for 72 h. The shoot heights and root lengths (cm) were measured with a tape measure.

#### Physio-biochemical parameters

##### Photosynthetic pigments content

Carotenoids and chlorophyll (Chl) pigments were extracted using fresh *Z. mays* leaves (0.1 g) in cold acetone 85% (v/v). After centrifugation at 8000 rpm (MIKRO 200R Hettich zentrifugen, Germany) for 10 min, the supernatant was collected and the absorbance was measured at 663, 644, and 452.5 nm using UV-Vis spectrophotometry (RIGOL, Model Ultra-3660) [34]. The concentration of pigments (carotenoids, Chl a, and b) was calculated and expressed as mg/g Fwt using formulas described by Lichtenthaler and Wellburn [35].

##### Relative water content (RWC) and membrane stability index (MSI) assessment

RWC was determined utilizing the technique outlined by Das et al. [36]. At first, the uppermost leaves of the plants were gathered and weighed instantly to determine their Fwt. Each leaf was then cut into 1 cm sections and immersed in distilled water for 12 h to achieve complete turgidity and the turgid weight (Twt) was measured. These leaf segments were then dried for 36 h at 72 °C in an oven to obtain their Dwt. Finally, the RWC was calculated using the following formula:

$$\:RWC\left(\%\right)=\frac{(Fwt-Dwt)}{(Twt-Dwt)}\times\:100$$

To evaluate the MSI of maize under both controlled and Pb-stressed conditions, the Farooq and Azam [37] technique was employed. About 1.00 g of leaves were prepared as thin pieces, which were then washed and kept for 4 h at 40 °C in a 30 mL of double-distilled water. The electrical conductivity (EC1) of the discs was measured using the electrical conductivity meter (Oakton Instruments, USA). The electrical conductivity (EC2) was measured after the sample discs were immersed in boiling water at 100 °C–30 min, without being taken out. The MSI was determined using the following formula:

$$\:\mathrm{M}\mathrm{S}\mathrm{I}\:=\:[1\:-\left(\frac{EC1}{EC2}\right)]\:\times\:100$$

##### Oxidative stress markers

The membrane leakage (ML) of maize leaves under Pb stress was assessed according to the methods of Quiroga et al. [38]. Firstly, the surfaces of leaf samples were cleaned by deionized water to remove any electrolytes. To measure the initial EC (Lo), the leaf samples were placed in closed vials with 25 mL of deionized water and incubated for 3 h at 25 °C in a shaker at 100 rpm. Subsequently, the vials were heated at 120 °C for 20 min in a water bath and cooled to 25 °C, and the final EC (Lf) was obtained.

The formula for ML is expressed as follows:

$$\:ML=\left[\right(Lo-Lw)/(Lf-lw\left)\right]\:\times\:100$$

*Lw represents the water’s conductivity used for sample incubation.

Lipid peroxidation was quantified by measuring the accumulation of malondialdehyde (MDA) via the thiobarbituric acid (TBA) reaction as established by Heath and Packer [39]. Fresh *Z. mays* leaf tissue (0.1 g) was homogenized at 4 °C in 2 mL of 0.1% trichloroacetic acid (TCA) using a mortar and pestle with fine granular quartz. The resulting homogenate was centrifuged at 12,000 rpm for 10 min at 4 °C. Next, 1 mL of the supernatant was thoroughly mixed with 4 mL of a reagent mixture comprising 20% TCA and 0.5% TBA. The mixture was boiled for 20 min at 95 °C and rapidly cooled to stop the reaction. The mixture was then centrifuged at 5000 rpm for 6 min. The absorbance was recorded at 532 nm. At 600 nm, the nonspecific absorption value was removed. The concentration of the MDA-TBA complex was then determined using an extinction coefficient of 155 mM^−1^cm^− 1^.

Hydrogen peroxide (H_2_O_2_) estimation was done according to Velikova et al. [40] protocol. Fresh *Z. mays* leaf samples (0.1 g) were homogenized in an ice bath with 3 mL of 0.1% (w/v) TCA. After the centrifugation at 12,000 rpm for 15 min, 0.5 mL of the resulting supernatant was mixed with 0.5 mL of 50 mM potassium phosphate buffer (PPB) (pH 7.5) and 1 mL of 1 M potassium iodide (KI). The absorbance was measured at 390 nm.

##### Total soluble protein and osmolytes contents

Fresh *Z. mays* leaves (0.5 g) were crushed in 5 mL of 50 mM PPB pH 7.0 for measuring the total soluble protein content. After that, the resulting mixture was centrifuged at 8000 rpm for 15 min at 4 °C. One mL of protein sample was combined with a newly made alkaline copper solution, left for 10 min, and then Folin-Ciocalteau reagent was added for 30 min. The absorbance was then recorded at 700 nm [41]. The concentration of total soluble proteins was calculated and expressed as mg/g Fwt using bovine serum albumin (BSA) as a standard.

Proline content was assessed using Bates et al. [42] technique. *Z. mays* leaves (0.25 g Fwt) were extracting and centrifuging in 3% sulfosalicylic acid. Then, 2 mL of the extract was reacted with 2 mL of ninhydrin reagent and 2 mL of glacial acetic acid and then placed in a boiling water bath for 60 min. After cooling, 4 mL of toluene was added, the upper layer was separated, and its absorbance was read at 520 nm.

Grieve and Grattan [43] protocol was used to assess the glycine betaine content of fresh maize leaves. *Z. mays* tissues were mixed with 1.5 mL of 2 N H_2_SO_4_ and heated in a water bath at 60 °C for 10 min, then combined with 50 µL cold KI-I_2_. The mixture was centrifuged for 15 min at 4 °C after being kept at 0–4 °C for 16 h. After removing the supernatant, 4.5 mL of 1, 2-dichloroethane was added to the precipitate. The absorbance was recorded at 365 nm after 2 h of room temperature incubation.

The total carbohydrate content was measured using the colorimetric phenol-sulfuric acid method [44]. Firstly, 2.5 N HCl was used to extract 100 mg of dried *Z. mays* leaves. Next, 1 mL of phenol (5%) and 5 mL of concentrated H_2_SO_4_ were mixed with 1 mL of the extract, and then the absorbance of the resulting solution was recorded at 490 nm. The carbohydrates’ final concentration was expressed as mg/g Dwt using a glucose standard curve.

##### Secondary metabolites (Total flavonoid [TFC] and phenolic [TPC]) contents

After crushing a known Fwt of *Z. mays* roots with ethanol (95%), the TFC was estimated by the AlCl_3_ colorimetric method [45] using a quercetin standard curve and it was calculated as µg quercetin equivalent (QE)/g Fwt. After centrifuging, the supernatant was collected, and 5 mL of distilled water was added, followed by adding 0.7 mL of 5% NaNO_3_ and 0.6 mL of 10% AlCl_3_. After that, at 510 nm, the absorbance was measured in comparison to a blank.

The TPC of *Z. mays* plants was assessed [46]. 0.5 mL of the ethanol extract was added to 1.4 mL of distilled water and 0.1 mL of 50% Folin-Ciocalteu phenol reagent. Next, 0.4% sodium carbonate was added. The absorbance was recorded at 650 nm. Using gallic acid as the standard, the TPC was measured as mg of gallic acid equivalent (GAE)/g Fwt.

##### Antioxidant enzyme activity

One gram of *Z. mays* fresh leaves under various treatments was extracted by grinding in 10 mL of 50 mM PPB (pH 7.0) comprising 0.1 mM EDTA (ethylenediaminetetraacetic acid) and 1% polyvinyl pyrrolidone [47]. After centrifuging the homogenate for 10 min at 4 °C at 12,000 rpm (Vision SCIENTIFIC CO., LTD., South Korea), the supernatant was gathered.

Peroxidase (POD) activity was measured at 470 nm according to the methods described by Chance and Maehly [48]. The reaction mixture was composed of 100 mM PPB (pH 7.0), 1% guaiacol, 0.4% H_2_O_2_, and 250 µL enzyme extract. The polyphenol oxidase (PPO) activity was determined according to the method of Beyer and Fridovich [49]. The reaction mixture involved 250 µL enzyme extract, 100 mM PPB (pH 7.0), and 100 µM pyrogallol. The absorbance was assessed at 430 nm, and the activity was calculated as U/g Fwt.

The Nakano and Asada [50] method was used to determine the ascorbate peroxidase activity (APX), which involved adding 250 µL of enzyme extract to a 3 mL reaction mixture containing 2.5 mL of 100 mM PPB (pH 7.0), 0.1 mL L-ascorbate, and 0.15 mL H_2_O_2_. The absorbance was measured at 290 nm compared to a blank, and its activity was computed using the ascorbate molar extinction coefficient (2.8 mM^− 1^ cm^− 1^).

#### Pb content in *Z. mays* plants

To assess the concentration of Pb in *Z. mays* tissues (shoots and roots), the samples were thoroughly rinsed with deionized water and oven-dried at 70 °C for 24 h. The dried tissues were pulverized into a fine powder using a mortar and pestle. A 0.5 g sample was digested in a 10 mL mixture of nitric acid (HNO_3_) and perchloric acid (HClO_4_) (3:1 v/v) overnight to ensure that the plant tissue was completely degraded. The next day, the samples were heated on a hot plate until the digest turned clear [51]. After filtration and dilution to 50 mL with deionized water, Pb concentrations were measured via atomic absorption spectrophotometer (AAS, Unicam 969) at the Faculty of Veterinary Medicine, Zagazig University. All measurements were performed in five independent replicates (*n* = 5). For the calculation of translocation factor (TF), Pb concentration in plant shoots was divided by Pb concentration in roots [52].

### Statistical data analysis

The experiments were conducted as a fully replicated factorial design with five independent biological replicates per treatment combination (*n* = 5). The variables were compared across treatments using SPSS software (Version 16.0, SPSS Inc., Chicago, IL, USA), and the means of each treatment were examined using one-way ANOVA and Duncan’s multiple range tests (DMRT) at the 95% probability level. The data were expressed as mean ± standard error (SE). Pearson correlation and clustering analysis were constructed between different measured physiological and biochemical parameters using the Past program version 4.3 to relate all measured parameters.

## Results

### Assessment of the in vitro tolerance of *T. harzianum* Zag-1 to Pb exposure

**Fig. 1:**
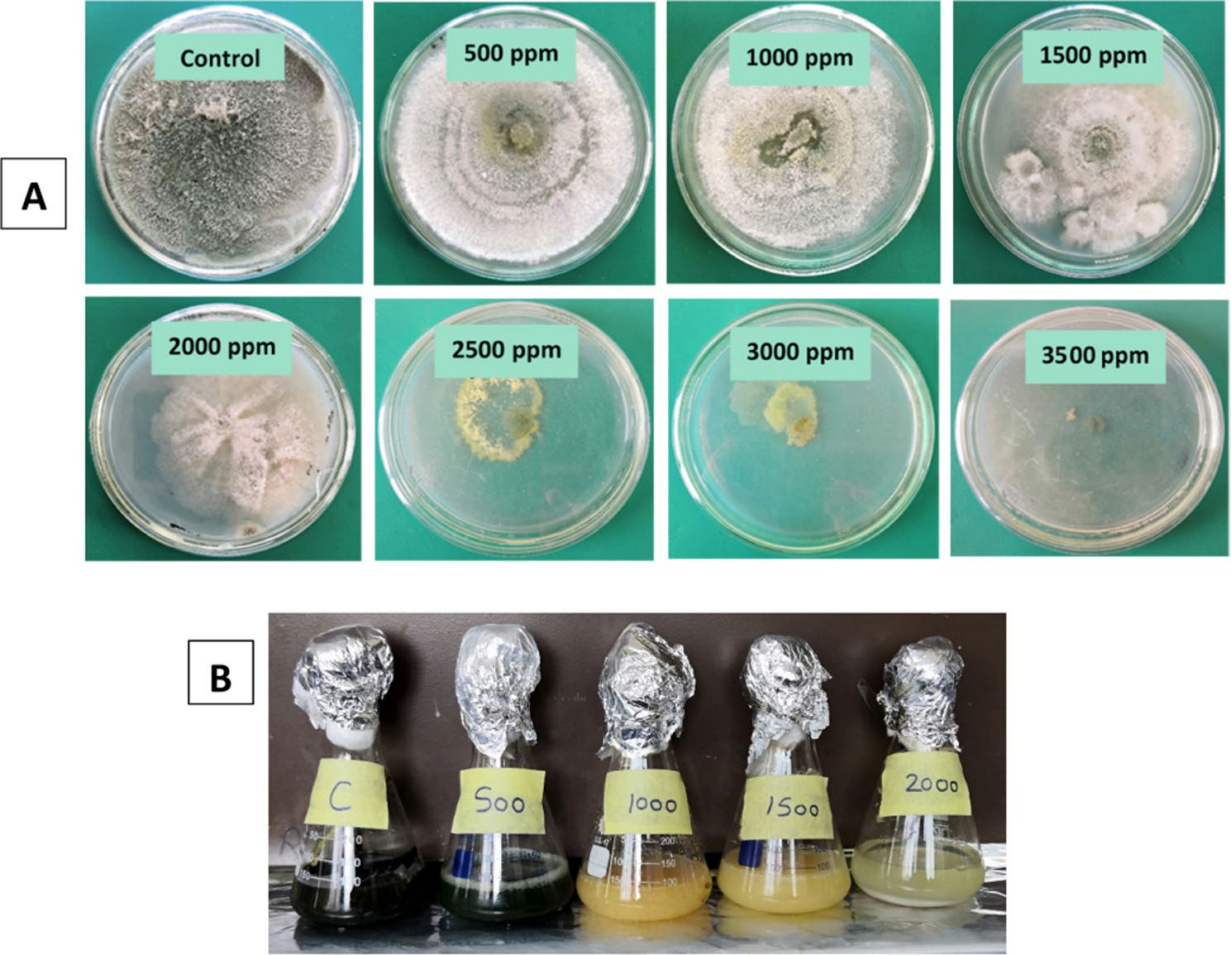
Effect of different Pb (II) concentrations on the growth parameters of *T. harzianum* Zag-1 including radial growth (**A**) on PDA and mycelia dry weight (**B**) on PDB

**Fig. 2:**
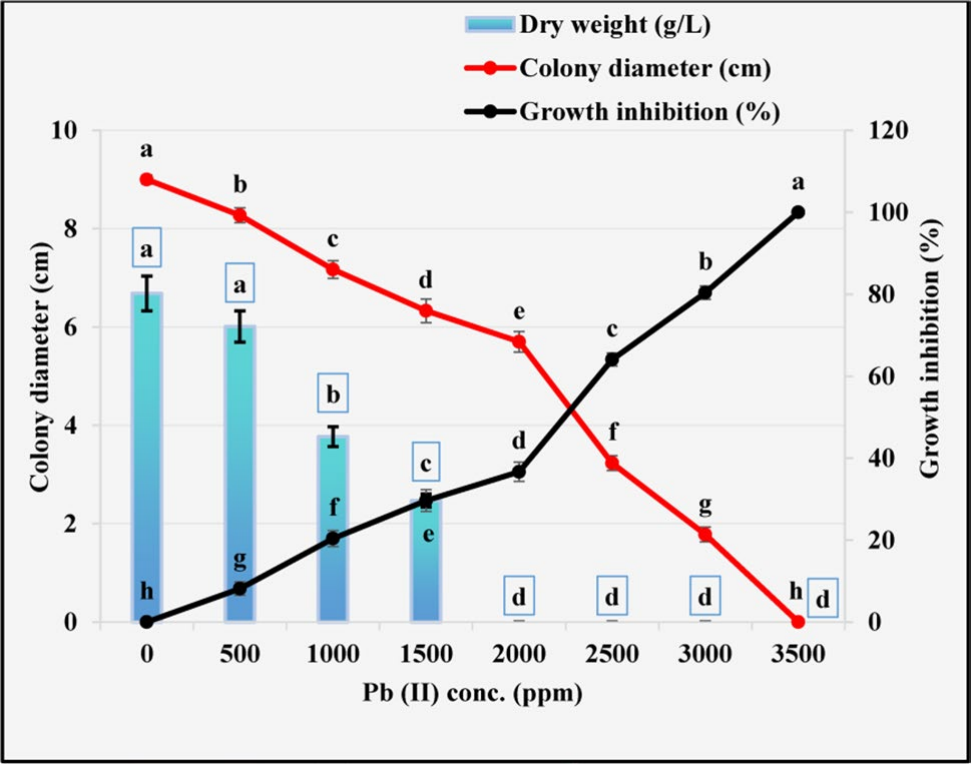
Effect of different Pb concentrations on the colony diameter (cm), growth inhibition (%), and mycelial dry weight (g/L) of *T. harzianum* Zag-1. *Data represent the mean of 5 replicates with standard error. Different letters indicate significant differences among treatments using a one-way ANOVA followed by the Duncan’s multiple range tests (*p* < 0.05)

Influence of different Pb (II) concentrations on linear growth and mycelial Dwt was presented in Figs. 1 and 2. Generally, the *T. harzianum’*s colony diameter decreased gradually by increasing Pb (II) concentration (Fig. 1A). The growth reduction percent ranged from 8.11% (500 ppm) to 80.22% (3000 ppm) as compared to the control (Fig. 2). *T. harzianum* continued their growth till concentration of 3500 ppm at which there is a complete growth inhibition. The biomass was slightly decreased by 10.03% at 500 ppm, while at concentrations of 1000 and 1500 ppm, the fungal growth was progressively diminished by 43.56 and 63.02%, respectively, as compared to the control (Figs. 1B and 2), while no growth was detected at 2000 ppm.

### Pb (II) removal and bioaccumulation by *T. harzianum* Zag-1

*T. harzianum* was assessed for its capacity to remove Pb (II) from PDB media supplemented with different concentrations of Pb (II) (Table 1). The bioremoval results showed that the Pb removal efficiency of *T. harzianum* was 70.40, 67.00, and 49.60% from the liquid media when the Pb (II) concentration was 500, 1000 and 1500 ppm, respectively. The maximum Pb (II) removal efficiency was 70.40%. However, the Pb (II) removal decreased with a further increase in its concentration.

**Table 1:** Pb removal efficiency (%) and uptake (Q) (mg/g) of *T. harzianum* Zag-1 under varied Pb (II) concentrations

Pb (II) conc. (ppm)	Pb Removal (%)	Pb Uptake (mg/g)
500	70.40 ± 3.725a	5.86 ± 0.310c
1000	67.00 ± 3.545a	17.75 ± 0.939b
1500	49.50 ± 2.619b	30.16 ± 1.595a

*Data represent the mean of 5 replicates ± standard error. Different letters indicate significant differences among treatments using a one-way ANOVA followed by the Duncan’s multiple range tests (*p* < 0.05)

Pb (II) bioaccumulation potential of *T. harzianum* was illustrated in Table 1. *T. harzianum* displayed greater Pb (II) levels with increasing concentrations of Pb (II) in the liquid media. This increment was particularly significant when comparing the results at 500 ppm with those at 1500 ppm, as *T. harzianum* showed a 5.15-fold increase in Pb(II) bioaccumulation. The results showed a negative relationship between the biomass production of fungus and the Pb (II) uptake by *T. harzianum*.

### Phenotypic traits

**Fig. 3:**
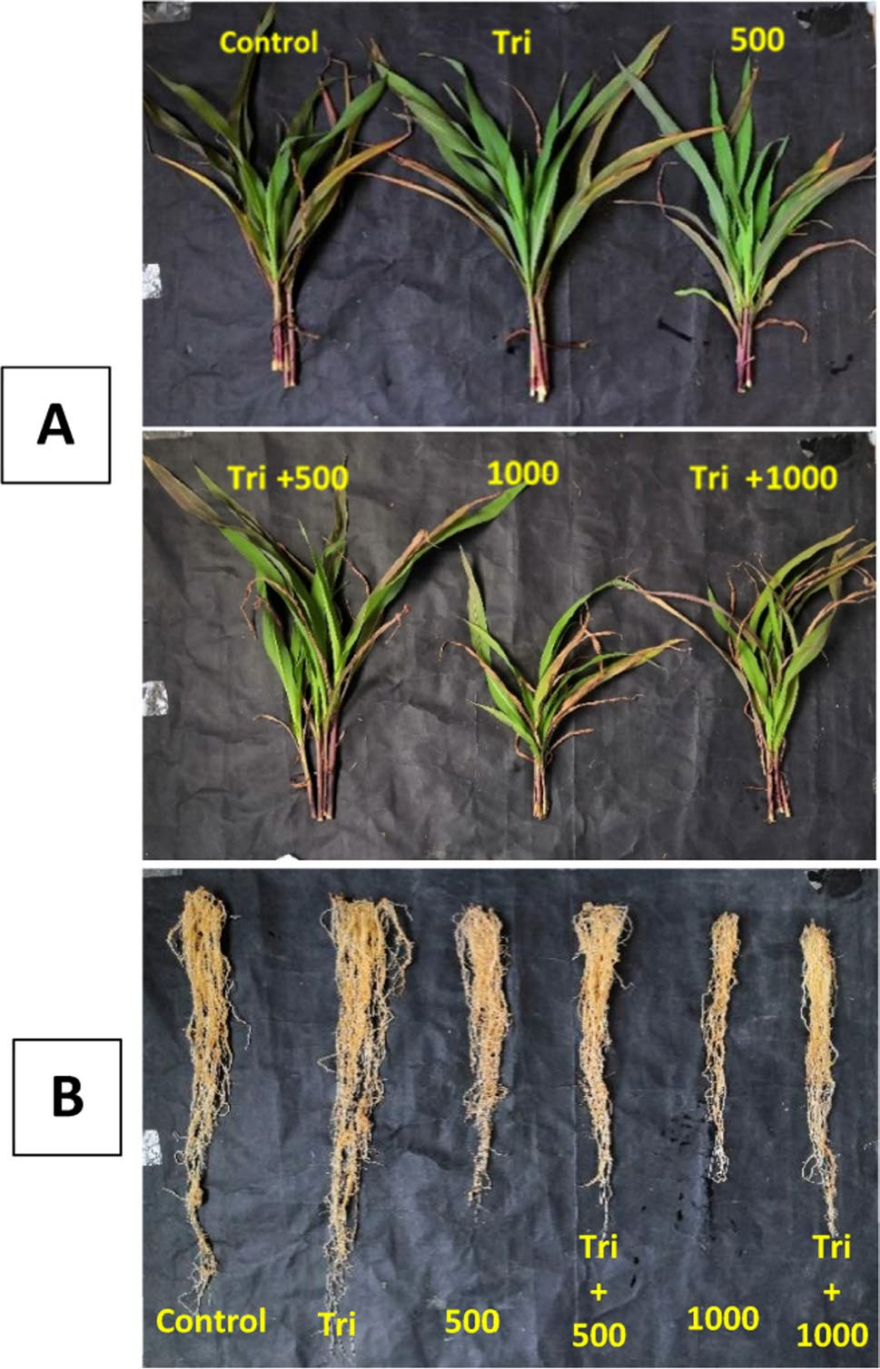
Effect of *T. harzianum* Zag-1 inoculation on the phenotypic traits [shoots (**A**) and roots (**B**)] of maize plants under Pb stress

The morphological characteristics of maize plants grown under Pb stress in response to *T. harzianum* Zag-1 inoculation are illustrated in Table 2; Fig. 3 (A and B). The findings showed that Pb stress resulted in significant reductions in growth metrics including shoot height and root length, Fwt and Dwt of the shoot and root system. The Pb stress (1000 ppm) lowered the shoot height and root length by 13.04 and 28.17%, respectively, in contrast to non-stressed maize plants. However, *T. harzianum* inoculation greatly improved these metrics under control and Pb stress conditions relative with the non-inoculated plants. *T. harzianum* Zag-1 inoculation raised maize shoot height and shoot Fwt and Dwt by 28.26, 18.33, and 8.95%, respectively, besides root length, root Fwt and Dwt by15.49, 17.33, and 33.33%, respectively, as compared with control plants. Under Pb (1000 ppm) stress, *T. harzianum* inoculation mitigated the Pb-negative impacts on shoot length, shoot Fwt and Dwt of maize plants by 12.5, 14.61, and 35.89%, respectively, and on root length, root Fwt and Dwt by 15.69, 16.02, and 7.14%, respectively, in contrast to non-inoculated plants.

**Table 2:** Effect of *T. harzianum* Zag-1 inoculation on phenotypic traits of maize plants grown under Pb stress conditions

Treatments	Shoot height (cm/plant)	Root length (cm/plant)	Shoot Fwt (g/plant)	Root Fwt (g/plant)	Shoot Dwt (g/plant)	Root Dwt (g/plant)
Control	46.00 ± 2.434bc	35.50 ± 1.878ab	5.29 ± 0.280b	3.23 ± 0.170b	0.67 ± 0.035a	0.33 ± 0.017c
*T. harzianum*	59.00 ± 3.121a	41.00 ± 2.169a	6.26 ± 0.331a	3.79 ± 0.200a	0.73 ± 0.039a	0.44 ± 0.023a
Pb (500 ppm)	43.00 ± 2.275bc	30.00 ± 1.59bc	4.35 ± 0.230cd	2.98 ± 0.158bc	0.46 ± 0.024cd	0.34 ± 0.018bc
*T. harzianum* + Pb (500 ppm)	49.00 ± 2.592b	35.50 ± 1.878ab	4.95 ± 0.261bc	3.31 ± 0.175ab	0.56 ± 0.029b	0.39 ± 0.021ab
Pb (1000 ppm)	40.00 ± 2.116c	25.50 ± 1.349c	3.49 ± 0.185e	2.56 ± 0.135c	0.39 ± 0.021d	0.28 ± 0.015c
*T. harzianum* + Pb (1000 ppm)	45.00 ± 2.381bc	29.50 ± 1.560c	4.00 ± 0.211de	2.97 ± 0.157bc	0.53 ± 0.028bc	0.30 ± 0.016c

*Data represent the mean of 5 replicates ± standard error. Different letters indicate significant differences among treatments using a one-way ANOVA followed by the Duncan’s multiple range tests (*p* < 0.05)

### Photosynthetic traits

The effect of Pb stress and *T. harzianum* Zag-1 inoculation on Chl content was presented in Table 3. Chl a + Chl b, carotenoids, and total pigment of maize plants were considerably reduced under Pb stress (1000 ppm) by 50.81, 58.62, and 52.75%, respectively, compared to non-stressed ones. On the contrary, the application of *T. harzianum* substantially increased all photosynthetic measurements under controlled and stressful conditions. Under controlled conditions, Zag-1 inoculation significantly raised the concentration of photosynthetic pigments compared to non-inoculated maize plants. This elevation was 69.77% for Chl a, 52.63% for Chl b, 64.52% for Chl a + Chl b, 22.41% for carotenoids, and 51.09% for total pigment. Under Pb stress (1000 ppm), *T. harzianum* Zag-1 inoculation boosted carotenoids and total pigment by 62.50 and 46.51%, respectively, in comparison to non-inoculated plants growing under the same Pb stress conditions.

**Table 3:** Effect of *T. harzianum* Zag-1 inoculation on chlorophyll (Chl) content (mg/g Fwt), carotenoids (mg/g Fwt), and total pigments (mg/g Fwt) of maize plants grown under Pb stress conditions

Treatments	Chl a	Chl b	Chl a + Chl b	Carotenoids	Total pigments
Control	0.86 ± 0.045b	0.38 ± 0.019b	1.24 ± 0.065b	0.58 ± 0.030b	1.82 ± 0.096b
*T. harzianum*	1.46 ± 0.077a	0.58 ± 0.030a	2.04 ± 0.107a	0.71 ± 0.037a	2.75 ± 0.145a
Pb (500 ppm)	0.47 ± 0.024cd	0.24 ± 0.012d	0.71 ± 0.037cd	0.25 ± 0.013e	0.96 ± 0.051d
*T. harzianum* + Pb (500 ppm)	0.93 ± 0.049b	0.42 ± 0.022b	1.36 ± 0.071b	0.49 ± 0.025c	1.84 ± 0.097b
Pb (1000 ppm)	0.41 ± 0.021d	0.20 ± 0.010d	0.61 ± 0.032d	0.24 ± 0.012e	0.86 ± 0.045d
*T. harzianum* + Pb (1000 ppm)	0.57 ± 0.0300c	0.30 ± 0.015c	0.87 ± 0.046c	0.39 ± 0.020d	1.26 ± 0.066c

*Data represent the mean of 5 replicates with standard error. Different letters indicate significant differences among treatments using a one-way ANOVA followed by the Duncan’s multiple range tests (*p* < 0.05)

### Water status and MSI

**Fig. 4:**
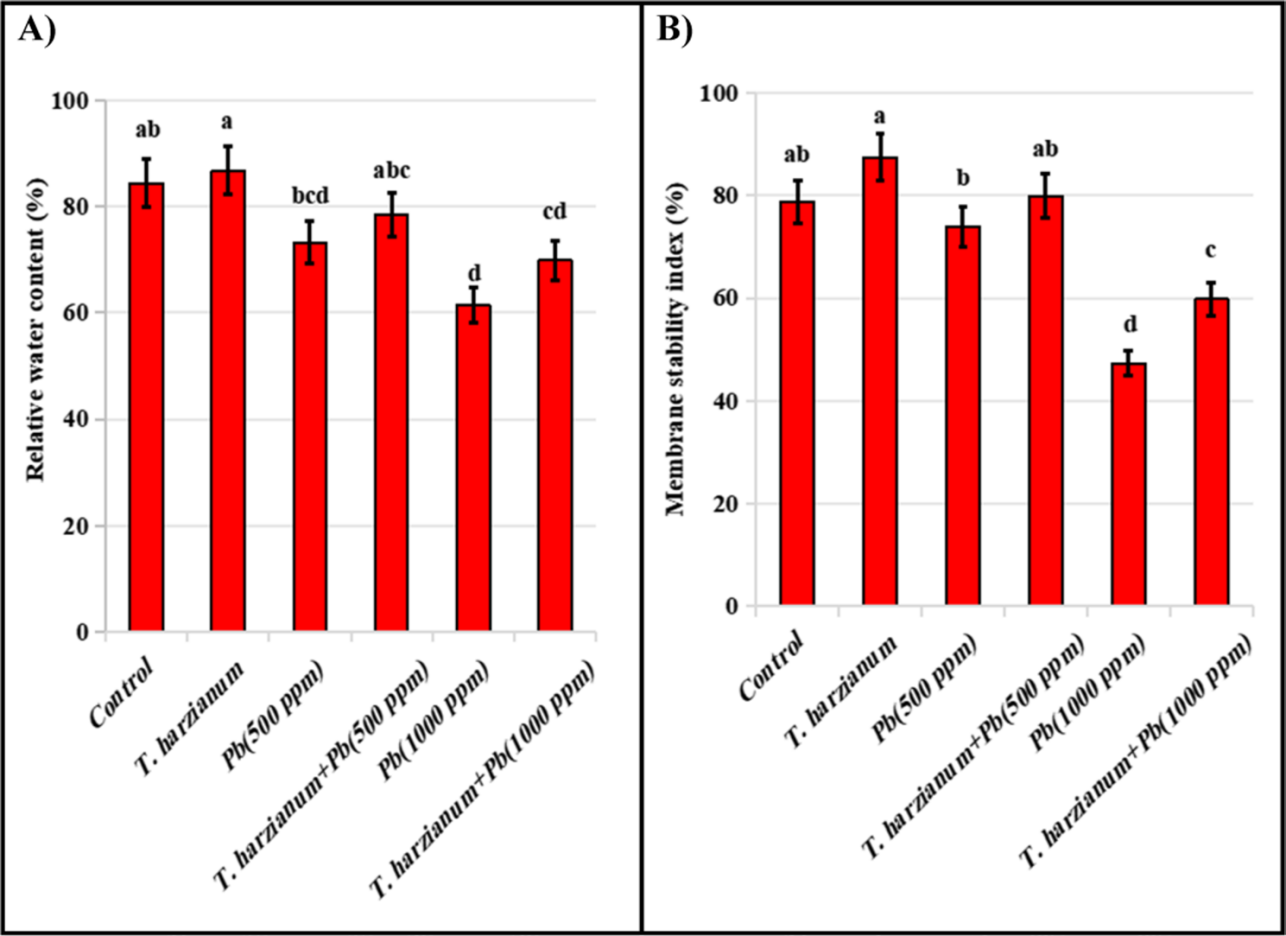
Impact of *T. harzianum* Zag-1 inoculation on (**A**): relative water content (RWC) and (**B**): membrane stability index (MSI) of maize plants grown under Pb stress conditions. *Data represent the mean of 5 replicates with standard error. Different letters indicate significant differences among treatments using a one-way ANOVA followed by the Duncan’s multiple range tests (*p* < 0.05)

The effect of *T. harzianum* Zag-1 and Pb stress on RWC and MSI was presented in Fig. 4. Pb stress either at 500 and 1000 ppm declined the RWC in contrast to the control; the maximum reduction was noted at 1000 ppm stress conditions in non-inoculated plants, with reduction of 27.26% compared to its corresponding controls. Although, at 500 and 1000 ppm Pb stress, *T. harzianum* Zag-1-inoculated plants showed greater RWC than their non-inoculated equivalents; the percent of increase was 7.06 and 13.84%, respectively, as compared to non-inoculated controls. A similar effect was noted for MSI, which remained significantly elevated in non-stressed plants irrespective of *T. harzianum* condition (Fig. 4). *T. harzianum* Zag-1 inoculation improved the MSI parameter by 8.04 and 26.23% in contrast to non-inoculated plants under 500 and 1000 ppm Pb stress, respectively.

### Oxidative stress markers

**Fig. 5:**
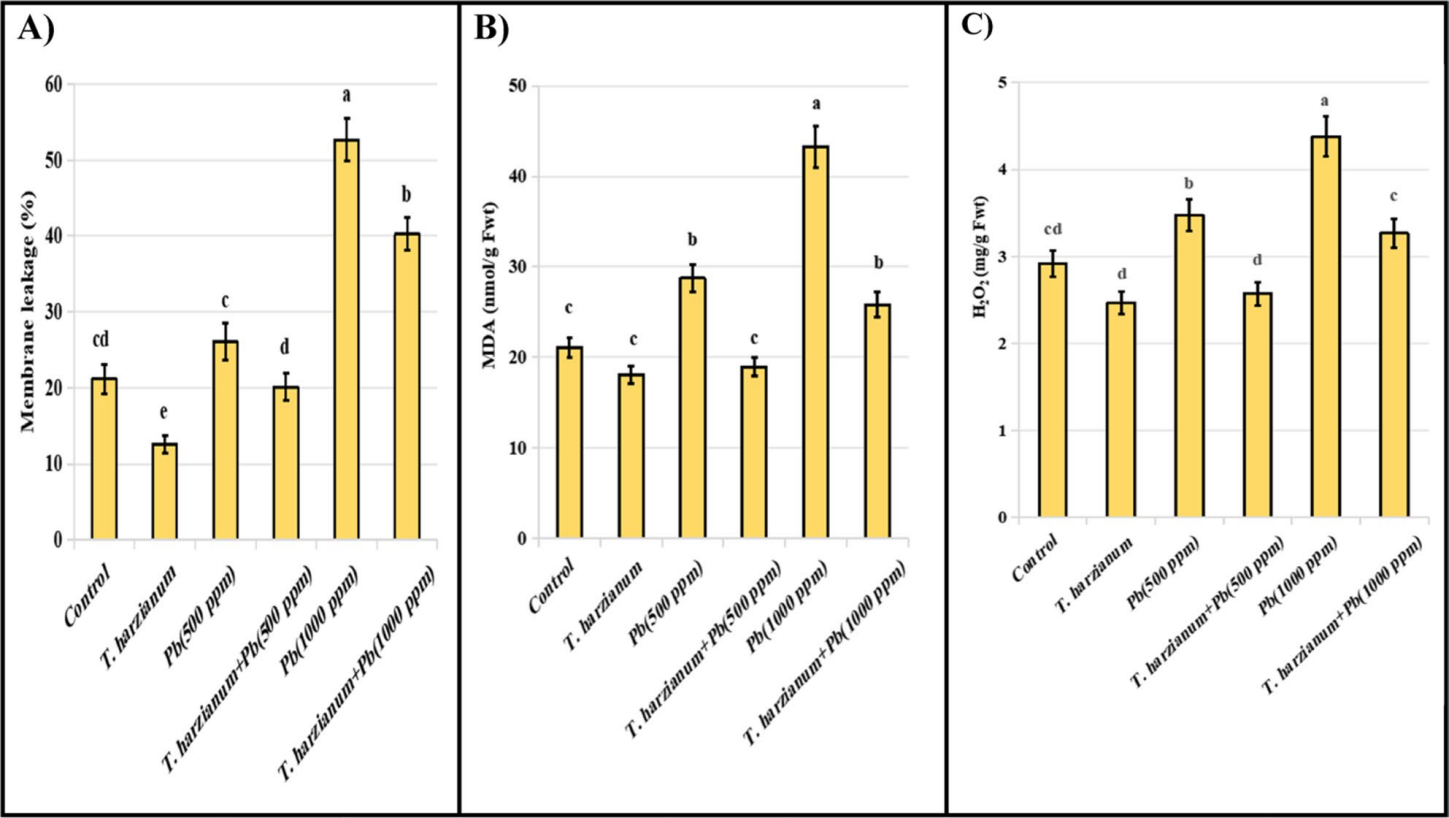
Impact of *T. harzianum* Zag-1 inoculation on oxidative stress markers: **A** membrane leakage (ML; %), **B** lipid peroxidation; (Malondialdhyde [MDA]) (nmol/g Fwt), and **C** H_2_O_2_ content (mg/g Fwt) of maize plants grown under Pb stress conditions. *Data represent the mean of 5 replicates with standard error. Different letters indicate significant differences among treatments using a one-way ANOVA followed by the Duncan’s multiple range tests (*p* < 0.05)

The level of oxidative stress indicators (ML, MDA, and H_2_O_2_ level) in Pb-stressed maize plants was depicted in Fig. 5. The data showed that all oxidative stress indicators gradually raised at both Pb levels, with the highest values at the high Pb level (1000 ppm) as shown in Fig. 5. Pb (500 ppm) significantly increased the ML, MDA, and H_2_O_2_ accumulation in maize leaves (22.98, 36.33, and 19.08%, respectively) as compared to their respective non-stressed controls. Conversely, under 1000 ppm Pb exposure, *T. harzianum* Zag-1 application led to a notable decrease in in ML, MDA, and H_2_O_2_, reaching 23.59, 40.36, and 25.41%, respectively, in comparison to the controls.

### Protective molecules in response to Pb stress

A remarkable change in total soluble protein and osmolytes content in *T. harzianum*-inoculated maize plants was substantial under both controlled and Pb-stressed conditions (Table 4). Pb stress (1000 ppm) resulted in significant accumulations of total soluble protein protein (1.78 mg/g Fwt), proline (12.42 µmols/g Fwt), and glycine betaine (13.15 mg/g Fwt) in contrast to the non-stressed plants (1.08 mg/g Fwt, 2.03 µmols/g Fwt, and 8.37 mg/g Fwt, respectively). On the contrary, a notable reduction in soluble carbohydrates was detected under stress. Most obviously, under 1000 ppm Pb, Zag-1-inoculated maize plants induced a further increase in soluble protein (12.36%), proline (45.65%) and glycine betaine (4.56%) compared to their corresponding controls. Under non-stressed condition, *T. harzianum* Zag-1 inoculation boosted carbohydrate levels (7.66%) and lessened Pb adverse impact (1000 ppm) by 37.65% compared to non-inoculated group.

**Table 4:** Effect of *T. harzianum* Zag-1 inoculation on total soluble proteins and osmolytes (proline, soluble carbohydrates and glycine betaine) contents of maize grown under Pb stress conditions

Treatments	Total soluble protein (mg/g Fwt)	Proline (µmols/g Fwt)	Soluble carbohydrates (mg/g Dwt)		Glycine betaine (mg/g Fwt)
Control	1.08 ± 0.060**d**	2.03 ± 0.098**e**	370.00 ± 20.681**a**	8.37 ± 0.442**d**	
*T. harzianum*	1.38 ± 0.076**c**	4.61 ± 0.235**d**	398.33 ± 22.180**a**	8.92 ± 0.470**cd**	
Pb (500 ppm)	1.61 ± 0.088**bc**	5.35 ± 0.274**d**	178.33 ± 10.539**cd**	10.43 ± 0.550**bc**	
*T. harzianum* + Pb (500 ppm)	1.74 ± 0.095**ab**	9.32 ± 0.484**c**	273.33 ± 15.566**b**	11.08 ± 0.585**b**	
Pb (1000 ppm)	1.78 ± 0.097**ab**	12.42 ± 0.648**b**	141.66 ± 8.599**d**	13.15 ± 0.694**ab**	
*T. harzianum* + Pb (1000 ppm)	2.00 ± 0.109**a**	18.09 ± 0.948**a**	195.00 ± 11.421**c**	13.75 ± 0.726**a**	

*Data represent the mean of 5 replicates ± standard error. Different letters indicate significant differences among treatments using a one-way ANOVA followed by the Duncan’s multiple range tests (*p* < 0.05)

### Secondary metabolites of maize plants in response to Pb stress

**Fig. 6:**
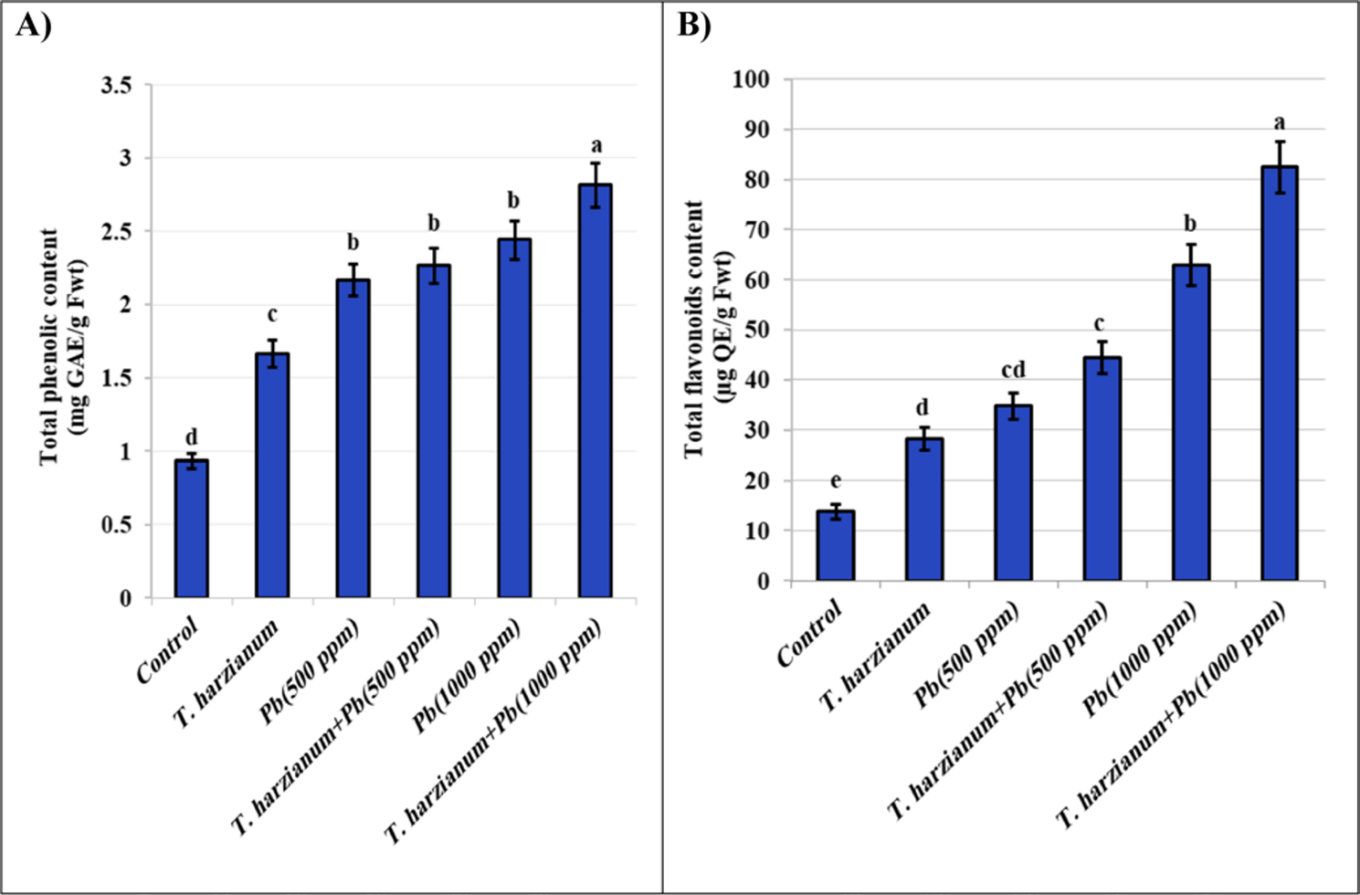
Impact of *T. harzianum* Zag-1 inoculation on secondary metabolites: **A** total phenolic content (mg GAE/g Fwt) and **B** total flavonoids content (µg QE/g Fwt) of maize plants grown under Pb stress conditions. GAE: Gallic Acid Equivalent, QE: Quercetin Equivalent. *Data represent the mean of 5 replicates with standard error. Different letters indicate significant differences among treatments using a one-way ANOVA followed by the Duncan’s multiple range tests (*p* < 0.05)

The influence of *T. harzianum* Zag-1 inoculation and Pb stress on secondary metabolites (TPC and TFC) was appeared in Fig. 6. *T. harzianum* inoculation boosted these metabolites’ levels by 78.04 and 104.00%, respectively, in comparison to their respective controls. The TPC and TFC content is increased by 2.61 and 4.56-fold in Pb-stressed (1000 ppm) plants in contrast to the non-stressed plants. However, under Pb stress (1000 ppm), *T. harzianum* Zag-1 inoculation showed an additional improvement in TPC (1.15-fold) and TFC (1.31-fold) in maize plants relative to non-inoculated ones.

### Impact of *T. harzianum* Zag-1 inoculation on antioxidant enzymatic responses of Pb-stressed maize

**Fig. 7:**
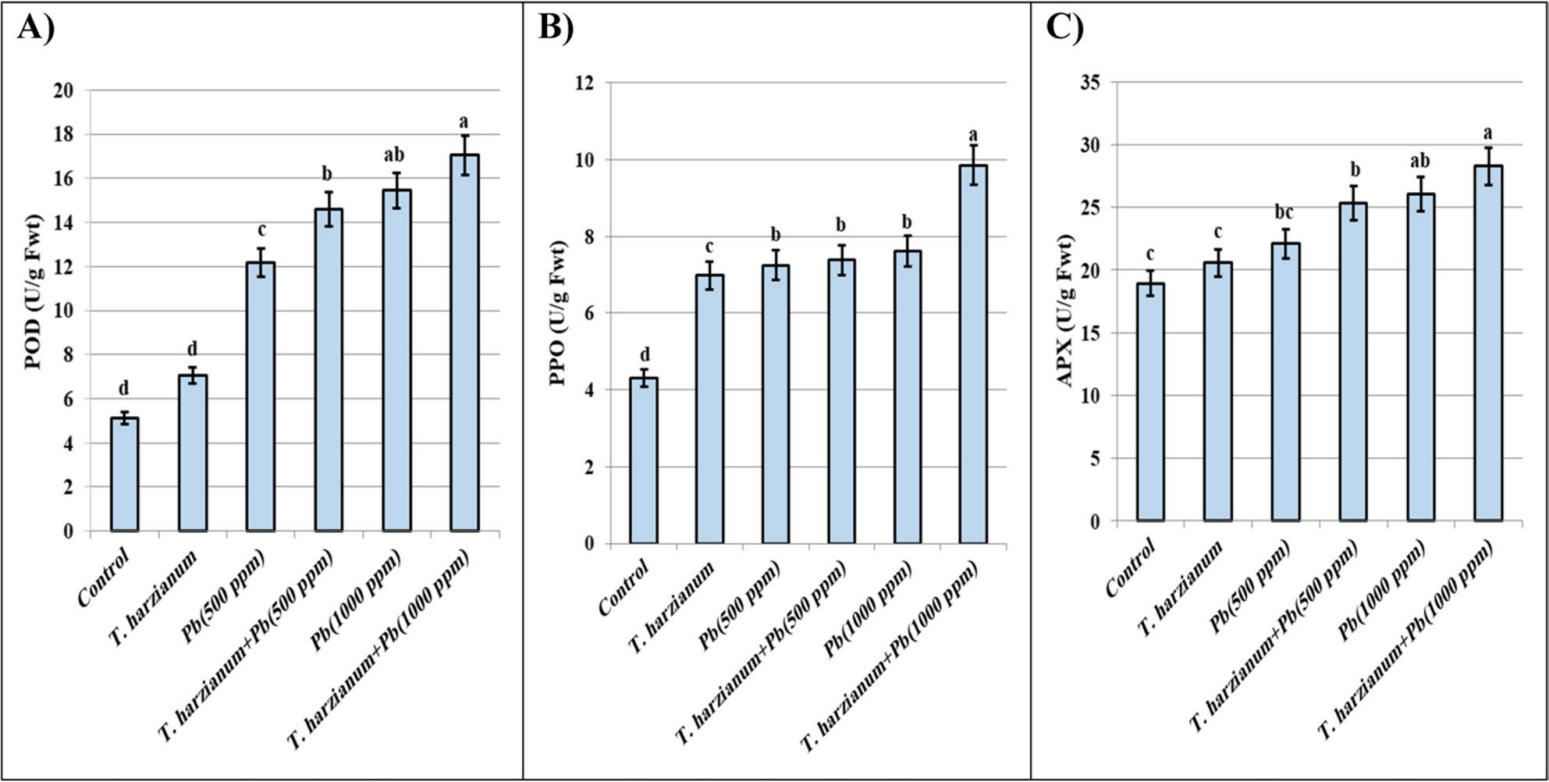
Impact of *T. harzianum* Zag-1 inoculation on antioxidant enzymes: **A** Peroxidase (POD), **B** Polyphenol oxidase (PPO) and **C** Ascorbate peroxidase (APX) in the shoots of maize plants under Pb stress conditions. *Data represent the mean of 5 replicates with standard error. Different letters indicate significant differences among treatments using a one-way ANOVA followed by the Duncan’s multiple range tests (*p* < 0.05)

The effect of *T. harzianum* Zag-1 inoculation on antioxidant enzymes activity such as POD, PPO, and APX under Pb stress was investigated in Fig. 7; their activities were significantly impacted by *T. harzianum* inoculation. Also, both Pb concentrations enhanced their activities. The activity of POD, PPO, and APX were 5.15, 4.31, and 18.94 U/g Fwt, respectively, in maize plants under controlled conditions, which maximally increased to 15.45 U/g Fwt (200%), 7.63 U/g Fwt (77.03%), and 26.07 U/g Fwt (37.65%), under Pb (1000 ppm) stress. Fascinatingly, *T. harzianum* Zag-1 caused considerably (*p* < 0.05) further increase in their activities in Pb-stressed maize. For instance, under Pb (1000 ppm), the activity of POD, PPO, and APX in *T. harzianum*-inoculated maize plants increased by 10.36, 29.22, and 8.44%, respectively, as compared to non-inoculated ones.

### *T. harzianum* Zag-1 reduces Pb accumulation in the shoots of *Z. mays*

Data in Table 5 showed that Pb concentrations in *Z. mays* roots and shoots were significantly increased under Pb stress. At 500 and 1000 ppm of Pb, the metal content was higher in roots (365.00 and 527.50 ppm, respectively) and shoots (275.00 and 410.00 ppm, respectively) compared to the non-stressed control plants. Fascinatingly, following inoculation of Pb-tolerant *T. harzianum* to *Z. mays* plants under 1000 ppm Pb, Pb accumulation were significantly increased in their roots (782.50 ppm) and decreased in their shoots (290.00 ppm) by 48.34 and 29.27%, respectively, as compared to non-inoculated plants. Simultaneously, exposure to 1000 ppm Pb significantly elevated the TF in *Z. mays*, resulting in greater transport of Pb from roots to shoots (0.78). However, this translocation was markedly diminished (0.37) following the application of *T. harzianum* under the same stress level.

**Table 5:** Pb content in shoots and roots and translocation factor (TF) of maize plants upon 500 and 1000 ppm and application of *T. harzianum* Zag-1

Treatments	Shoot Pb content (ppm)	Root Pb content (ppm)	Translocation factor (TF)
Control	0d	0d	–
*T. harzianum*	0d	0d	–
Pb (500 ppm)	275.00 ± 14.551b	365.00 ± 19.313c	0.75
*T. harzianum* + Pb (500 ppm)	200.00 ± 10.583c	377.50 ± 19.975c	0.53
Pb (1000 ppm)	410.00 ± 21.695a	527.50 ± 27.912b	0.78
*T. harzianum* + Pb (1000 ppm)	290.00 ± 15.345b	782.50 ± 41.406a	0.37

*Data represent the mean of 5 replicates ± standard error. Different letters indicate significant differences among treatments using a one-way ANOVA followed by the Duncan’s multiple range tests (*p* < 0.05)

### Principal component analysis and hierarchical clustering analysis

**Fig. 8:**
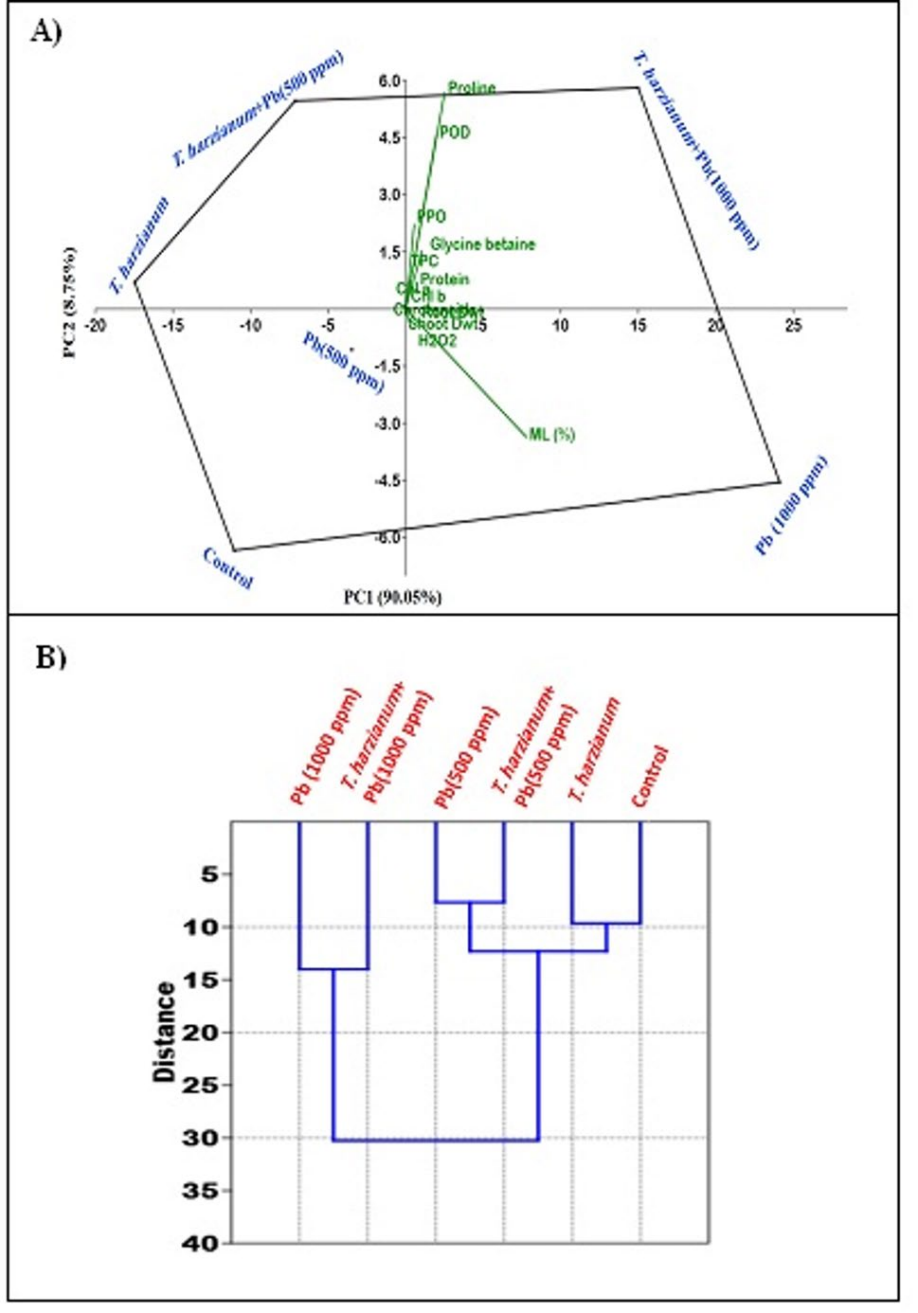
Principal component analysis (biplot) between the different treatments (blue) and the studied parameters (green) of *Z. mays* plants under the effects of *T. harzianum* Zag-1 and Pb-stress (**A**) and Hierarchical clustering analysis (**B**)

Principal component analysis (PCA) was conducted to assess the associations between the measured parameters and treatments with and without *T. harzianum* under Pb stress (500 and 1000 ppm) (Fig. 8A). The cumulative proportions of the first and second components (PC1 and PC2) accounted for 98.80% of the total variance in the dataset. PC1 accounted for 90.05% of the variance in the data that was acquired, whereas PC2 accounted for 8.75% of the total variation. The various PCA biplot parameters were most obviously divided into several clusters. Measures like ML and oxidative stress indicator (H_2_O_2_) were strongly separated and were close to the Pb-stress (1000 ppm) treatment and located on the opposite site of the plot from growth metrics and photosynthetic pigments, which were highest in the control and *T. harzianum* treatments. Crucially the application of *T. harzianum* to Pb-stressed plants shifted the physiological profile toward the accumulation of osmoprotectants such as proline and glycine betaine, as well as enhanced antioxidant enzyme activities (POD and PPO). This indicates that the inoculation of *T. harzianum* specifically helps the plant by boosting these defense mechanisms. The results of the main component analysis were corroborated by the hierarchical clustering analysis (HCA), which showed the differences between treatments (Fig. 8B).

### Pearson correlation matrix

**Fig. 9:**
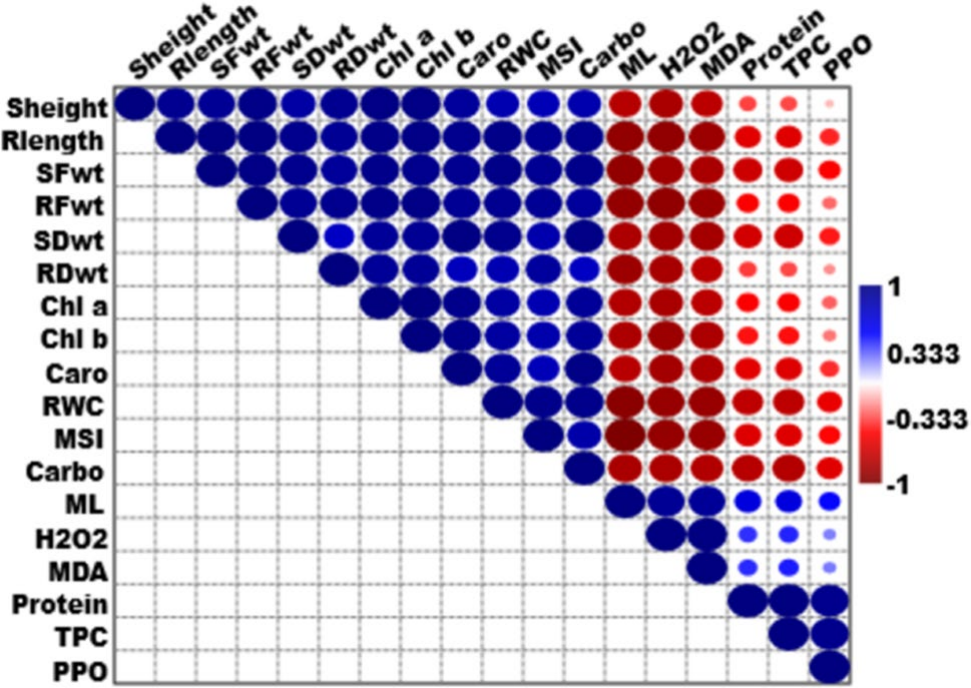
Pearson’s correlation matrix among morphological and biochemical parameters in *Z. mays* plants. The intensity of color ranges from blue (positive) to red (negative) and the size of the circles shows the strength of significant correlation (*p* < 0.05). Sheight: Shoot height, Rlengh: Root length, SDwt: Shoot dry weight, RDwt: Root dry weight, SFwt: Shoot fresh weight, RFwt: Root fresh weight, Caro: Carotenoid content, RWC: Relative water content, MSI: Membrane stability index, Carbo: Carbohydrates content, ML: Membrane leakage, H_2_O_2_: Hydrogen peroxide, MDA: Malondialdehyde, TPC: Total phenolic content, PPO: Polyphenol oxidase

**Fig. 10:**
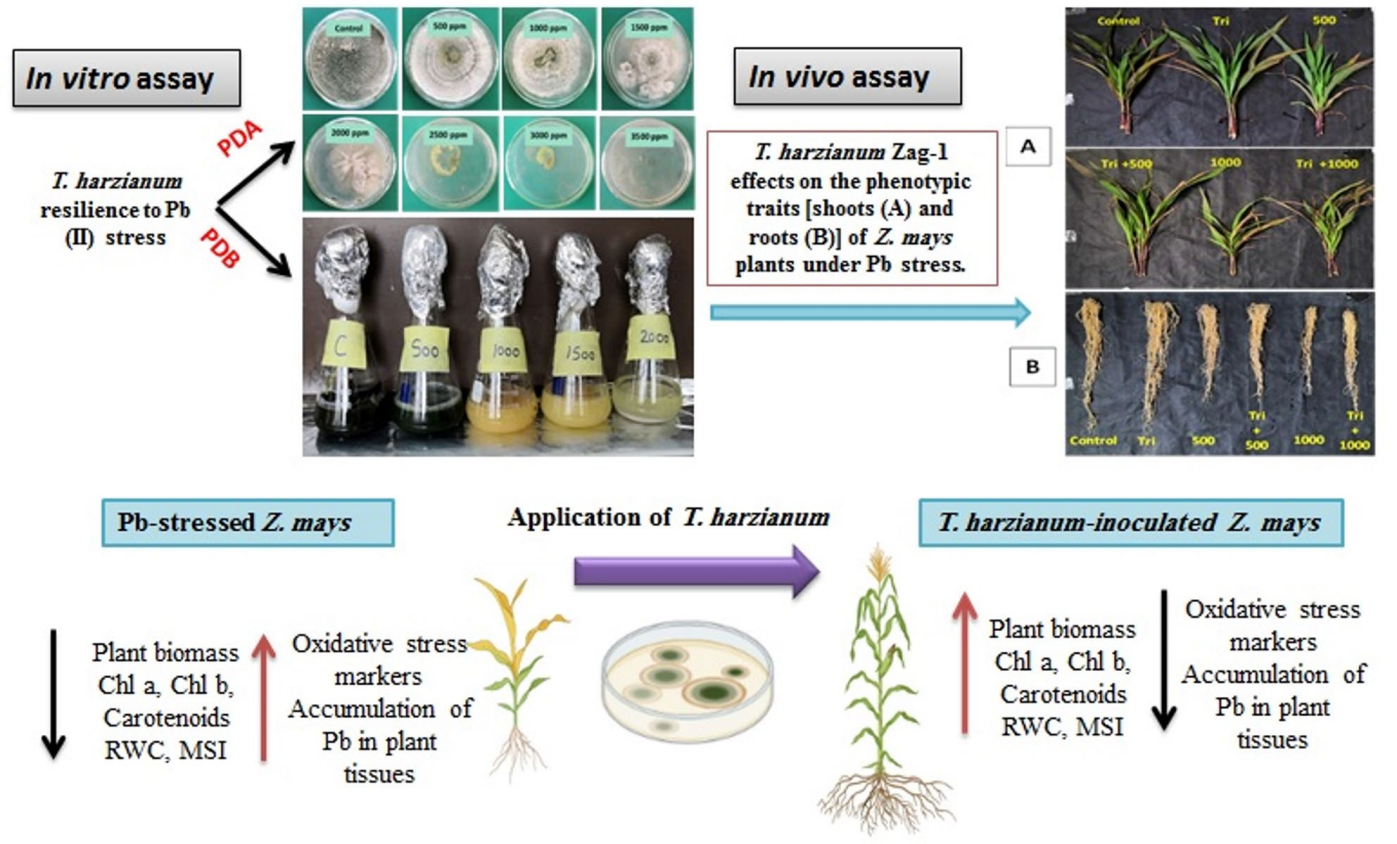
Schematic overview and key results of maize plants after treatment with Pb and *T. harzianum* Zag-1. Increase is indicated in red arrow and decrease is indicated in black arrow. RWC: Relative water content and MSI: Membrane stability index

A Pearson correlation matrix was conducted to assess the correlation between morphological and biochemical attributes, as depicted in Fig. 9. Significant positive or negative correlations were found for most of the traits under study. A strong positive correlations (deep blue color) were found between morphological traits (shoot and root parameters), photosynthetic pigments (chl a, chl b, and carotenoids), RWC, and MSI. This strong correlation suggests that the application of *T. harzianum* improved the plant’s chlorophyll, resulting in optimized plant growth performance. In contrast, all growth parameters, chl a, chl b, carotenoids, RWC, and MSI had negative correlations with oxidative stress indicators (ML, H_2_O_2_ and MDA). These markers were elevated in Pb-stressed plants, highlighting the oxidative damage accompanied with Pb-induced stress. However, a noticeable decline in MDA and H_2_O_2_ accumulation was found in *T. harzianum*-treated plants, resulting in reduction in cellular damage as also represented in Fig. 10 which shows a schematic overview of the key results of maize plants after treatment with Pb and *T. harzianum *Zag-1.

## Discussion

To satisfy the ever increasing worldwide food requirements, it is crucial for developing crop varieties that are more resilient to a variety of environmental conditions, such as HMs contamination that has developed as a major hazard [53–55]. Sustainable techniques must be used to address these harmful pollutants in order to achieve the best possible plant development and yield [56, 57]. The potential of *T. harzianum* Zag-1 for improving Pb tolerance in maize plants cultivated in Pb (II)-amended soils was examined. HM tolerant fungi, such as *Aspergillus welwitschiae* [58] and *Trichoderma* sp [59] have been addressed for their essential roles in enhancing crops and in HM remediation. In our investigation *T. harzianum* Zag-1 could tolerate Pb(II) up to 1500 ppm on PDB medium. Similarly, Malik et al. [60] reported that Pb-tolerant endophyte *Trametes hirsute* could grow up to 1500 mg L^− 1^ of Pb (II). In contrast to our results, Hoseinzadeh et al. [61] found that *T. harzianum* TS103 tolerate only up to 200 mg L^− 1^ Pb. Moreover, the increased Pb (II) concentrations caused growth suppression of *T. harzianum* Zag-1 mycelial biomass, which might influence protein synthesis and the activity of enzymes and also induce cell death [62]. Furthermore, Malkoc et al. [63] and Metwally and Abdelhameed [5] reported that high HM concentrations exert drastically stress on the fungal strain, inhibiting growth as a result of reactive oxygen species (ROS) accumulation. The results indicated a maximum Pb (II) removal efficiency of 70.40% at 500 ppm, followed by a concentration-dependent decline as the initial Pb (II) concentration was further increased. Similar results have been obtained with *Phanerochaete chrysosporium* [64] under Pb stress. The reduction in metal removal efficiency with an increase in concentration has been reported [65, 66], and it may be due to enhancement in competition of metal ions with available binding sites on the fungal biomass. At higher concentration, most of the metal ions did not accumulate by the fungus might be due to saturation of fungal biomass [65]. The results illustrated a negative correlation between the growth and uptake of Pb (II) by *T. harzianum*. Several reports supported our findings [67, 68]. Similar results were reported by Yaghoubian et al. [69]. They found that growth of *Trichoderma* sp. and Cd(II) uptake were negatively correlated. Additionally, Joshi et al. [70] stated that Pb uptake by *A. terreus* from industrial wastewater was because of the existence of more binding sites in cell wall. Both of passive processes, such biosorption onto the cellular surface, which is the initial method used in interaction with pollutants, and active processes, like intracellular sequestration, are involved in the bioaccumulation of metal ions from the environment [71, 72].

In our study, the remarkable reduction in all morphological attributes of maize plants, including length, Fwt, and Dwt of both shoot and root systems (Table 2; Fig. 3) as a result of Pb stress (500 and 1000 ppm) might be related to its phytotoxicity, induced lipid peroxidation, and impairment of vital plant processes [5, 73]. Similarly, an earlier study reported a significant reduction of *Brassica campestris* L. plants’ growth in soil contaminated with HMs [74]. Zrig et al. [75] reported that antimony (Sb) exposure significantly repressed maize productivity; decreasing Fwt and Dwt, while arbuscular mycorrhizal fungi (AMF) addition considerably mitigated its toxicity, improving Fwt and Dwt. However, *T. harzianum* Zag-1 inoculation resulted in an improvement of all measured morphological metrics under both normal and Pb-stressed conditions due to its great potential in stimulating root absorption and development, promoting stress resistance, and boosting soil microecology [31, 76]. The application of *Trichoderma* sp. can positively influence plant biomass by promoting the indole-3-acetic acid (IAA) availability, a hormone that stimulates root growth and elongation, leading to more efficient nutrient absorption [77]. In addition, *Trichoderma* can increase plant height, thereby enhancing plant biomass, as observed in research by Yadav et al. [29] on tomato seedlings exposed to high levels of HMs. Our results align with Hussain et al. [78] found that the application of *A. niger* to Pb-stressed maize plants reconstructed the length of root and shoot and plant Fwt and Dwt. The growth of roots favors uptake of water and nutrients and mitigates adverse abiotic impacts, which may have a positive effect on biomass accumulation in bean plants inoculated with *Trichoderma* [79]. Moreover, Hazimaha et al. [80] reported that the inoculation of *Trichoderma* sp. to red chilli (*Capsicum annuum* L.) cultivated under Al stress improved plant height and its Dwt. Additionally, comparable results were found in plants exposed to different abiotic circumstances, for instance, salinity, cold temperatures, drought, and excess water [23, 81–83]. Furthermore, Altaf et al. [30] proved that *Trichoderma* sp. TF-13 inoculation lowered HM-caused toxicity in *Vigna radiata* L. due to its capacity to detoxify metals and raise plant root Fwt and Dwt.

Photosynthesis is a crucial sign of healthy plant growth and development; it supports regular biochemical, physiological, and molecular activities within plant cells [84]. Chl content is a major factor in determining a plant’s capability to perform photosynthesis and plant health [85]. In our investigation, the Chl and carotenoids concentrations in maize plants were significantly impacted by *T. harzianum* inoculation and Pb stress. A reduction in photosynthetic pigments under abiotic stress caused by the breakdown of enzymes (RuBP Carboxylase and ATP synthase). These enzymes play a vital role in the electron transport chain (ETC) and Calvin cycle, and their breakdown results in thylakoid membranes and stomatal conductance damage [86]. Also, Pb affects the Chl, carotenoids, and plastoquinone biosynthesis as well as reduces the activity of C3 cycle enzymes; consequently, photosynthesis is slowed down [87]. Moreover, HMs’ toxicity causes ROS generation, which results in photo-oxidative injury. Consistent with our results, Altaf et al. [30] showed that total Chl and carotenoid levels of *V. radiata* leaves were dramatically decreased under metal stress. Oppositely, *Trichoderma*-inoculated maize plants exhibited a substantial increase in Chl and carotenoids content due to their involvement in allowing less Ca^2+^ and Mg^2+^ to leach from soil and increasing their uptake, with special consideration of Mg^2+^ which forms a vital part of Chl [88]. Similarly, the leaf pigment content of Cd-treated chickpea plants was positively impacted by single and dual treatments of *Trichoderma sp.* with *Pseudomonas fluorescence* [57]. Likewise, Sun et al. [89] demonstrated that the application of Pb-tolerant *T. asperellum* SD-5 improved *Lolium perenne* L. growth and leaf Chl content under Pb stress. Abdelrhim et al. [90] and Saha et al. [91] demonstrated that *T. koningii* and *T. harzianum* inoculation lessened abiotic stress in *Phaseolus vulgaris* and Indian mustard and boosted the Chl content.

In the current investigation, RWC and MSI, two physiological parameters that have a direct connection to water uptake by plant roots and water loss via transpiration, drastically dropped in maize leaves subjected to Pb stress (500 and 1000 ppm) as opposed to non-stressed plants (Fig. 4), which might be associated with Pb-induced stomatal closure that limits water absorption [92, 93]. HMs affect water transport to the shoot by inhibiting transpiration as they reduce leaf size, lamina thickness, intercellular spaces, number of stomata, and their opening [94]. Additionally, increased Pb levels injure cellular membranes, causing stomatal closure, which results in CO_2_ deficit and impaired water relations [87, 95]. However, *T. harzianum* inoculation improved RWC and MSI due to its boosted capability to extract water from the rhizosphere [96] and regulate stomatal opening, thus lowering water loss [97]. Similarly, the increment of leaf RWC in plants inoculated with *T. atroviride* and *T. asperellum* has been found in *Z. mays* plants under abiotic stress [96, 98]. In addition, enhanced pigment production under *T. harzianum* Zag-1 inoculation might have restored the plant’s photosynthetic capacity, leading to more water absorption followed by increased RWCs under Pb stress [99].

Even though the oxidative stress markers (ML, MDA, and H_2_O_2_) steadily rose in maize leaves with increasing Pb levels, the highest values were obtained at 1000 ppm of Pb stress (Fig. 5). ROS overproduction are crucial signaling molecules, which result in oxidative stress [100], that can seriously harm membranes by elevating lipid peroxidation and oxidation of DNA and proteins in plants [23, 101]. Also, due to the effect of HMs on plant cell walls, the membranes become more permeable, and the level of electrolyte leakage (ML) is raised [102]. The increased H_2_O_2_ accumulation in agronomically important plant species such as *Z. mays* [103], *Cajanus cajan* [104], *Persicaria hydropiper* [105], and *Sorghum bicolor* [106] exposed to different metals had been reported. Muradoglu et al. [107] observed that MDA content was significantly increased in strawberry under Cd stress. Amusingly, *T. harzianum* inoculation led to a substantial reduction in MDA and H_2_O_2_ levels and improved the MSI compared to Pb-stressed plants, reflecting alleviation of oxidative stress [108, 109] and the protection of membranes as an obvious consequence of inoculation with microorganisms.

Enzymatic and non-enzymatic antioxidants in *T. harzianum-*inoculated maize plants lower lipid peroxidation of the plasma membrane and control cellular homeostasis [110] and reduce ML that might help in maintaining membrane integrity, possibly through reducing Pb bioavailability within plant tissues. Similarly, saprophytic fungal consortium and *Rhizophagus irregularis*, singly or in dual inoculation, potentially minimized HM-caused toxicity in *Solanum lycopersicum* L. grown under higher levels of HMs and markedly reduced the MDA level [111]. HMs increased MDA and proline levels in *Triticum aestivum* L.; however, these contents decreased with metal-tolerant endophytic fungus *Penicillium roqueforti* inoculation [112]. Moreover, Altaf et al. [30] and Zhang et al. [85] proved that *Trichoderma* sp. TF-13 and *T. nigricans* T32781 reduced Pb and Cd effects and decreased the amount of MDA and H_2_O_2_ in metal-stressed *V. radiata* L. and *Nicotiana tabacum* L. The measurement of MDA, H_2_O_2_ and ML data revealed that non-Zag-1 inoculated plants contain considerably higher levels of these compounds than inoculated ones. This demonstrates that *T. harzianum* Zag-1 inoculation decreased Pb-induced oxidative stress on maize plants, causing an enhanced response to Pb stress.

In order to maintain regular physiological and metabolic processes, plants produce and remove ROS in a balanced manner. Stressed plants have an imbalance that leads to increased ROS production, cell viability loss, and membrane collapse, all of which promote oxidative damage [57, 113]. Typically, when plants face HM stress, the activity of enzymatic and non-enzymatic antioxidants significantly increases as a self-defense response [114]. Osmoregulatory compounds such as proline, glycine betaine, amino acids, and total soluble carbohydrates are produced in a plant’s cytoplasm under both favorable and adverse conditions [55]. Lead stress activated proline, protein, and glycine betaine synthesis in maize. Proline helps effectively reduce the oxidative damage caused by ROS and is required to maintain cellular osmolality under high metal exposure; it serves as a chaperone, maintaining the structure of cytoplasmic proteins, and its accumulation adjusts the pH, preserves the cell’s redox status, and offers optimal NADP+/NADPH ratios proper for metabolism [115, 116]. Also, glycine betaine is essential for lowering the generation of ROS, helps recover photosynthesis, preserves membranes, and is crucial for the integrity of components, such as ribulose-1,5-bisphosphate carboxylase/oxygenase, and protein complex structures [117]. Similarly, proline exhibited Se tolerance in *P. vulgaris* [118]. On the contrary, the ability of Pb stress to reduce carbohydrates in maize plants may be caused by an excess of abscisic acid, which prolongs the closure of plant stomata, inhibits photosynthesis, reduces the synthesis of soluble sugars, and lowers plant biomass [101].

Interestingly, *T. harzianum* inoculation resulted in higher levels of osmolytes, such as sugars, in maize plants compared to non-inoculated plants that may regulate the plants’ metabolism of carbohydrates in response to HMs stress, probably by changing the plants’ energy distribution, resulting in maintaining cell membrane permeability and osmotic potential and boosting abiotic stress resilience in a variety of plants [119–122]. *T. harzianum* Zag-1 inoculation may also stimulate proline biosynthetic activity to control osmotic pressure, maintain enzymes and cellular structures, and eliminate ROS [123]. For example, *T. longibrachiatum* boosted the accumulation of soluble sugar, proline, and soluble protein under abiotic stress in *Pinus massoniana* L. seedlings to preserve anatomical stability and minimize water loss [124]. Stressed plants typically upregulate the production of secondary metabolites, including flavonoids, phenolic acids, glucosinolates, and alkaloids [125, 126]. These molecules influence antioxidant activity by scavenging ROS and supporting the development of cell walls, bolstering the plant’s physical barriers [127]. Plants had accumulated an array of phenolic and flavonoid compounds to adapt to stress conditions, since these substances are essential antioxidants that shield plants from oxidative damage and increase their resistance to HMs’ toxicity [128]. In the present study, *T. harzianum* Zag-1 application led to more production of phenolics and flavonoids in maize plants under Pb stress (500 and 1000 ppm). Our findings are consistent with earlier research demonstrating that helpful inoculants can improve plant resistance by encouraging further increases in phenolic and flavonoid biosynthesis, which is frequently elevated under HMs stress as a defensive mechanism [129]. Similarly, dos Santos et al. [116] found a significant increase in phenols and flavonoids production in the plant-*Trichoderma* interaction under different abiotic stresses. Besides, in response to oxidative stress, *Trichoderma* accumulates phenolic compounds that are linked to the activation of the plant’s biochemical defense system [127]. Furthermore, Shalaby [130] found that garlic plants inoculated with *T. harzianum* enhanced phenolic and flavonoid compounds.

Furthermore, antioxidant enzymes such as superoxide dismutase (SOD), guaiacol peroxidase (GPX), CAT, POD, and APX could scavenge ROS, minimize crop damage, and boost stress resistance [131]. Our study showed that *T. harzianum* Zag-1 inoculation enhanced maize antioxidant systems by enhancing the production of POD, PPO, and APX, which diminished oxidative damage and resulted in a successful ROS scavenging that led to effective management of MDA. Azcón et al. [132] proposed that symbiotic fungal cells might mainly provide protection against HM-induced oxidative stress by facilitating the elimination of ROS from plant cells. Similarly, Jamal et al. [133] reported that inoculation of wheat plants with *A. Welwitschiae* enhanced antioxidative enzyme activities under Cr stress. Additionally, Hazimaha et al. [80] reported that the treatment with *Trichoderma* sp. effectively enhanced CAT and APX in *C. annuum* L. plants and countered the adverse effects of Al stress. POD is an essential alternate method of H_2_O_2_ elimination that can be found throughout the cell with a much greater affinity to H_2_O_2_ than CAT [134]. APX is a fundamental component of the ascorbate-glutathione (AsA-GSH) cycle, which is crucial for plants to scavenge ROS [135] and is found in the cytosol, cell wall, vacuole, and extracellular spaces and initiates the reduction of H_2_O_2_ to water by ascorbate [136]. Plant growth, development, and stress tolerance are all significantly influenced by PPO enzymes [137]. This enzyme shows broad-spectrum antioxidant abilities through its interactions with peroxidase systems and the water–water cycle [138]. The overproduction of TPC with Pb stress could be linked to the higher activity of POD, which catalyzes the formation of polyphenols and cell wall lignification [139].

Our results showed a significant increase in Pb accumulation in both the roots and shoots of *Z. mays* following exposure to 500 and 1000 ppm of Pb. Although Pb is a non-essential element, its transport from the soil to various plant organs is often facilitated by the synthesis of protons, root exudates, and other metabolites, resulting in metal solubilization via the formation of metal-chelating complexes. Consistent with our findings, Ansari et al. [2] found that the concentration of Pb in the shoot and root of *Lallemantia iberica* increased with increasing Pb levels. Conversely, interesting results were obtained with *Trichoderma*-treated plants where the *T. harzianum* supplementation decreased the uptake of Pb in *Z. mays* through decreasing its bioavailability within the rhizosphere. Our finding suggests that *T. harzianum* application limits the translocation of Pb to *Z. mays* shoots by sequestering the metal within the root system. Rhizospheric microorganisms have the capacity to limit the mobility of metals by entrapping them with cell−surface polymeric compounds, rendering them unavailable for plant uptake [30]. *Trichoderma* alleviates HMs’ toxicity on plants through multiple mechanisms, including valence state modification, intracellular/extracellular metal localization, and biosorption [140]. Furthermore, *Trichoderma* produces various bioactive polysaccharides and proteins that bind HM ions through electrostatic adsorption, complexation, chelation, and ion exchange [25]. These processes collectively reduce the fraction of Pb available for translocation to the aerial parts of *Z. mays*. Similar findings have been reported by Jam et al. [141], where *T. harzianum* inoculation effectively reduced the translocation of HMs to the aboveground tissues of hairy vetch (*Vicia villosa*) in Pb- and Zn- contaminated soils. Likewise, metal-resistant strains of *T. harzianum* decreased Cd concentrations in *Hordeum vulgare* by reducing the metal uptake [142]. Additionally, Altaf et al. [30] reported that *Trichoderma* sp. TF-13 significantly reduced Pb content in roots and shoots of *Vigna radiata*. More recently, Badri Abdulhadi Mohammed Al-Haidari et al. [143] observed that arbuscular mycorrhizal fungi (AMF) restricted Pb translocation from roots to shoots in ornamental plants.

The mitigation of Pb-induced oxidative stress by *Trichoderma* spp. is a multifaceted process involving both direct and indirect mechanisms. These fungi can reduce metal toxicity through biosorption, bioaccumulation, and biotransformation processes, in addition to secreting siderophores and organic acids that chelate Pb and decrease its bioavailability in the rhizosphere [59, 144, 145]. For instance, *T. asperellum* has been shown to produce oxalic acid and thiol-containing compounds that bind Pb ions, thereby limiting their reactivity and toxicity [146]. Moreover, fungal inoculation enhances plant antioxidant defense systems, including enzymatic and non-enzymatic components, leading to reduced oxidative damage and improved stress tolerance [85]. In parallel, siderophore production contributes to metal chelation and may indirectly modulate nutrient availability and plant signaling pathways associated with systemic resistance. The high in vitro Pb removal efficiency observed in this study is consistent with these mechanisms and is reflected in the reduced Pb accumulation in plant tissues under in vivo conditions, supporting the functional relevance of Zag-1 in mitigating Pb toxicity.

## Conclusion

*T. harzianum* Zag-1 application suggests a viable technique to mitigate the negative impacts of Pb stress (500 and 1000 ppm) on maize plants through the activation of the antioxidant defense system, which promotes the elimination of ROS, therefore shielding proteins and membranes from oxidative damage. This study proved that *T. harzianum* Zag-1 inoculation enhanced growth, physiological, biochemical, and photosynthetic characteristics, osmolytes, and secondary metabolites production in maize plants under different levels of Pb stress (500 and 1000 ppm). Furthermore, the study reveals that *T. harzianum* application resulted in a critical reduction in Pb translocation from the roots to the shoots of *Z. mays* plants. These findings highlight the promising potential of *T. harzianum* Zag-1 as a sustainable bio-fertilizer and bioremediation agent for Pb-contaminated soils to restore these contaminated agricultural soils and improve food security in HM-affected areas.

## Data Availability

Data will be made available on request.

## Abbreviations

APX: Ascorbate peroxidase
Dwt: Dry weight
DMRT: Duncan’s multiple range tests
EC: Electrical conductivity
Fwt: Fresh weight
GAE: Gallic acid equivalent
HM: Heavy metal
H_2_O_2_: Hydrogen peroxide
Pb: Lead
MDA: Malondialdehyde
ML: Membrane leakage
MSI: Membrane stability index
POD: Peroxidase
PPO: Polyphenol oxidase
PDA: Potato dextrose-agar
RWC: Relative water content
SE: Standard error
TBA: Thiobarbituric acid
TAC: Total antioxidant capacity
TFC: Total flavonoid content
TPC: Total phenolic content
TCA: Trichloroacetic acid
